# Guselkumab treatment of psoriasis results in sustained reduction of systemic inflammatory biomarkers to week 48 in three phase III randomized controlled trials

**DOI:** 10.1093/skinhd/vzag075

**Published:** 2026-06-15

**Authors:** Niamh Kearney, Miriam Zimmermann, Alianu Akawung, Brian Kirby

**Affiliations:** Department of Dermatology, Our Lady of Lourdes Hospital, Drogheda, Ireland; Johnson & Johnson, Zug, Switzerland; Johnson & Johnson Research & Development, LLC, Spring House, PA, USA; Department of Dermatology, St Vincent’s University Hospital Dublin, Dublin,Ireland; Charles Institute of Dermatology, University College Dublin, Dublin,Ireland

## Abstract

Increased risk of major adverse cardiac events (MACE) may be linked to systemic inflammation for patients with psoriasis. Guselkumab is an effective treatment for psoriasis and psoriatic arthritis that was previously shown to reduce inflammatory biomarkers in three phase 3 randomized controlled trials [to week 16 in VOYAGE 1 (A Study of Guselkumab in the Treatment of Participants with Moderate to Severe Plaque-Type Psoriasis)/VOYAGE 2 (A Study of Guselkumab in the Treatment of Participants with Moderate to Severe Plaque-Type Psoriasis With Randomised Withdrawal and Retreatment) and week 12 in ECLIPSE (A Study to Evaluate the Comparative Efficacy of CNTO 1959 (Guselkumab) and Secukinumab for the Treatment of Moderate to Severe Plaque-Type Psoriasis) ]. In this *post hoc* analysis, sustained reduction in neutrophil/lymphocyte ratio, platelet/lymphocyte ratio and monocyte/lymphocyte ratio were observed to week 48 in these three trials for patients who received guselkumab every 8 weeks. This reduction was independent of improvements in Psoriasis Area Severity Index scores. Observational studies are required to identify whether this reduction correlates with a reduction in MACE.

Dear Editor,

Patients with psoriasis are at increased risk for major adverse cardiac events (MACE), with the excess risk for those with severe psoriasis in the order of 70% for myocardial infarction (MI), 56% for stroke and 39% for cardiovascular death.^1^ MACE may be linked with psoriasis by virtue of systemic inflammation.^2^ The pathophysiology of psoriasis is dominated by the secretion of interleukin (IL)-17 by T-helper 17 (Th17) cells, with proliferation and activation of Th17 cells driven by IL-23.^2^ These inflammatory cytokines, in addition to tumour necrosis factor (TNF), are also implicated in the development of coronary atherosclerotic plaque, subsequent thrombosis and MI.^2^ In rheumatoid arthritis, treatment with TNF-α inhibitors reduces the incidence of MI, which could be related to reduced systemic inflammation.^3^ Neutrophil/lymphocyte ratio (NLR), platelet/lymphocyte ratio (PLR) and monocyte/lymphocyte ratio (MLR) are biomarkers of systemic inflammation and cardiovascular risk, and predict poorer outcomes in people with MACE.^4^

Guselkumab, a p19 subunit-targeted IL-23 inhibitor, is an effective treatment for chronic plaque psoriasis, psoriatic arthritis and inflammatory bowel disease. We have previously reported reductions in NLR, PLR and MLR with guselkumab at week 16 in the two phase III randomized controlled clinical trials of guselkumab, VOYAGE 1 (A Study of Guselkumab in the Treatment of Participants with Moderate to Severe Plaque-Type Psoriasis; NCT02207231) and VOYAGE 2 (A Study of Guselkumab in the Treatment of Participants with Moderate to Severe Plaque-Type Psoriasis With Randomised Withdrawal and Retreatment; NCT02207244), and at week 12 in the ECLIPSE trial [A Study to Evaluate the Comparative Efficacy of CNTO 1959 (Guselkumab) and Secukinumab for the Treatment of Moderate to Severe Plaque-Type Psoriasis; NCT03090100].^5^ The aims of this post hoc analysis were to evaluate NLR, PLR and MLR to week 48 in participants who received guselkumab every 8 weeks in these trials and to determine whether changes in them were independent of or dependent on disease severity. All participants who received guselkumab continuously from week 0 in the trials were included. Participants who received placebo or active comparator (i.e. adalimumab or secukinumab) and those who responded who were withdrawn from guselkumab treatment as per the VOYAGE 2 study protocol were not included. Statistical analysis was completed using SAS studio version 9.4 (SAS Institute, Cary, NC, USA). NLR was calculated as neutrophil count/lymphocyte count, PLR as platelet count/­lymphocyte count and MLR as monocyte count/lymphocyte count, with each presented as mean (SD). A Student’s paired *t*-test was used to compare each at baseline and week 48. Pearson’s correlation coefficient was used to measure the strength of the relationship between change in Psoriasis Area Severity Index (PASI) and change in NLR, PLR and MLR.

Baseline characteristics are as reported in the original publications for each study.^6–8^ Baseline NLR, PLR and MLR were similar between the groups in each trial at baseline.^5^ In VOYAGE 1, significant reductions in NLR from 2.66 to 2.38 (*P* < 0.001) and PLR (from 150.9 to 138.6; *P* < 0.001) were found from baseline to week 48, while a nonsignificant reduction in MLR (from 0.24 to 0.23; *P* = 0.59) was observed (Table 1). In VOYAGE 2, all three biomarkers, NLR (from 2.65 to 2.33; *P* < 0.001), PLR (from 152.7 to 143.6; *P* = 0.001) and MLR (from 0.25 to 0.23; *P* < 0.001), decreased significantly from baseline to week 48 (Table 1). Similarly, in ECLIPSE, significant reductions were seen for NLR (from 2.80 to 2.40; *P* < 0.001), PLR (from 146.4 to 133.4; *P* < 0.001), and MLR (from 0.26 to 0.23; *P* < 0.001) (Table 1). In VOYAGE 1 and VOYAGE 2, changes in NLR, PLR and MLR, while statistically significant, showed only weak correlations with changes in PASI (*r*-values ranging from −0.174 to −0.122). Our findings suggest no meaningful relationship between these measures with changes in NLR, PLR and MLR possibly being independent of changes in disease severity. No significant correlation between changes in NLR, PLR and MLR and changes in PASI was observed in ECLIPSE (Table 1).

**Table 1:** Comparison of neutrophil/lymphocyte ratio (NLR), platelet/lymphocyte ratio (PLR) and monocyte/lymphocyte ratio (MLR) at baseline and week 48, and correlation analysis of change in NLR, PLR and MLR compared with change in Psoriasis Area and Severity Index (PASI) in participants treated continuously with guselkumab in the phase III VOYAGE 1, VOYAGE 2 and ECLIPSE randomized controlled clinical trials

Comparison of NLR, PLR and MLR at baseline and week 48						
	*n*	Biomarker	Baseline	Week 48	Change^a^	*P*-value^b^
VOYAGE 1	300	NLR	2.66 (1.20)	2.38 (1.07)	−0.29 (−0.41, −0.16)	<0.001
	299	PLR	150.9 (55.4)	138.6 (49.1)	−12.3 (−17.3, −7.4)	<0.001
	300	MLR	0.24 (0.10)	0.23 (0.11)	−0.01 (−0.02, 0.0004)	0.59
VOYAGE 2	271	NLR	2.65 (1.15)	2.33 (1.16)	−0.33 (−0.46, −0.19)	<0.001
	269	PLR	152.7 (54.8)	143.6 (53.5)	−9.15 (−14.70, −3.60)	0.001
	271	MLR	0.25 (0.11)	0.23 (0.09)	−0.02 (−0.03, −0.01)	<0.001
ECLIPSE	499	NLR	2.80 (1.38)	2.40 (1.03)	−0.41 (−0.52, −0.30)	<0.001
	497	PLR	146.4 (55.9)	133.4 (46.0)	−12.96 (−16.87, −9.04)	<0.001
	499	MLR	0.26 (0.12)	0.23 (0.10)	−0.02 (−0.03, −0.01)	<0.001

Correlation analysis of change in NLR, PLR and MLR compared with change in PASI				
		Δ in NLR vs. Δ in PASI	Δ in PLR vs. Δ in PASI	Δ in MLR vs. Δ in PASI
VOYAGE 1	*n*	298	297	298
	Pearson’s *r*^c^	−0.174	−0.127	−0.122
	*P*-value^d^	0.003	0.029	0.035
VOYAGE 2	*n*	444	439	444
	Pearson’s r^c^	−0.157	−0.167	−0.150
	*P*-value^d^	<0.001	<0.001	0.001
ECLIPSE	*n*	498	496	498
	Pearson’s r^c^	−0.007	0.014	−0.015
	*P*-value^d^	0.879	0.756	0.738

NLR, PLR and MLR values presented as mean (SD). Δ, change. ^a^Difference from baseline to week 48 reported as mean (95% confidence interval). ^b^*P*-value calculated using Student’s paired *t*-test with comparison between baseline and week 48 values. ^c^Pearson’s correlation coefficient. ^d^Comparison of change in biomarkers compared with change in PASI between baseline and week 48.

We previously reported a reduction in systemic inflammation induced by guselkumab at week 12 in VOYAGE 1 and VOYAGE 2, and at week 16 in ECLIPSE, as measured using NLR, PLR and MLR, and that these observations were independent of improvements in disease severity. The present analysis of participants treated with guselkumab from baseline to week 48 demonstrates that this reduction in systemic inflammation is sustained and appears largely independent of changes in disease severity as evaluated by PASI.

NLR, PLR and MLR are biomarkers of systemic inflammation and MACE risk.^2^ Continued treatment with guselkumab alters these biomarkers and, consequently, may be associated with a reduced incidence of MACE. This may, in part, explain the reduced mortality reported in patients treated with systemic therapies.^9^ Earlier intervention with biologics, such as guselkumab, for psoriasis may not only improve the burden of skin disease, but also reduce systemic inflammation and potentially the risk of MACE.

## Author contributions

Niamh Kearney (Conceptualization, Formal analysis, Investigation, Methodology, Project administration, Visualization [equal], Writing—original draft [lead]), Miriam Zimmermann (Data curation, Formal analysis, Funding acquisition, Investigation, Methodology, Project administration, Resources, Software, Validation, Writing—review & editing [equal]), Alianu Akawung (Data curation, Formal analysis, Funding acquisition, Investigation, Methodology, Project administration, Resources, Software, Validation, Writing—review & editing [equal]), and Brian Kirby (Conceptualization, Formal analysis, Investigation, Methodology, Supervision, Writing—review & editing [equal])

## Data Availability

The data-sharing policy of Janssen Pharmaceutical Companies of Johnson & Johnson is available at https://www.janssen.com/clinical-trials/transparency. Requests for access to the study data can be submitted through the Yale Open Data Access (YODA) Project site.
